# Small RNA Regulators of Plant-Hemipteran Interactions: Micromanagers with Versatile Roles

**DOI:** 10.3389/fpls.2016.01241

**Published:** 2016-08-30

**Authors:** Sampurna Sattar, Gary A. Thompson

**Affiliations:** College of Agricultural Sciences, The Pennsylvania State UniversityUniversity Park, PA, USA

**Keywords:** sRNAs, hemiptera, resistance, RNAi, viral immunity

## Abstract

Non-coding small RNAs (sRNAs) in plants have important roles in regulating biological processes, including development, reproduction, and stress responses. Recent research indicates significant roles for sRNA-mediated gene silencing during plant-hemipteran interactions that involve all three of these biological processes. Plant responses to hemipteran feeding are determined by changes in the host transcriptome that appear to be fine-tuned by sRNAs. The role of sRNA in plant defense responses is complex. Different forms of sRNAs, with specific modes of action, regulate changes in the host transcriptome primarily through post-transcriptional gene silencing and occasionally through translational repression. Plant genetic resistance against hemipterans provides a model to explore the regulatory roles of sRNAs in plant defense. Aphid-induced sRNA expression in resistance genotypes delivers a new paradigm in understanding the regulation of *R* gene-mediated resistance in host plants. Unique sRNA profiles, including changes in sRNA biogenesis and expression can also provide insights into susceptibility to insect herbivores. Activation of phytohormone-mediated defense responses against insect herbivory is another hallmark of this interaction, and recent studies have shown that regulation of phytohormone signaling is under the control of sRNAs. Hemipterans feeding on resistant plants also show changes in insect sRNA profiles, possibly influencing insect development and reproduction. Changes in insect traits such as fecundity, host range, and resistance to insecticides are impacted by sRNAs and can directly contribute to the success of certain insect biotypes. In addition to causing direct damage to the host plant, hemipteran insects are often vectors of viral pathogens. Insect anti-viral RNAi machinery is activated to limit virus accumulation, suggesting a role in insect immunity. Virus-derived long sRNAs strongly resemble insect piRNAs, leading to the speculation that the piRNA pathway is induced in response to viral infection. Evidence for robust insect RNAi machinery in several hemipteran species is of immense interest and is being actively pursued as a possible tool for insect control. RNAi-induced gene silencing following uptake of exogenous dsRNA was successfully demonstrated in several hemipterans and the presence of *sid-1 like* genes support the concept of a systemic response in some species.

## Introduction

Small RNAs (sRNAs) are essential regulators of eukaryotic gene expression and function. These 20–30 nucleotide (nt) regulatory elements (Aravin et al., 2003), common to both plants and animals, control endogenous gene expression in response to external stimuli and protect the host from invasive viruses. Plants respond to changing environmental conditions by altering their transcriptome, which is actively modulated by sRNAs. Altered expression of sRNA and their gene targets, in response to abiotic and biotic stress have firmly established the importance of these regulatory elements. During biotic stress, plants identify the pathogen associated molecular patterns (PAMPs), which initiates a downstream signaling cascade leading to PAMP-triggered immunity (PTI). Pests and pathogens have simultaneously evolved effector proteins to halt PTI and launch effector-triggered susceptibility (ETS). Plants have co-evolved to acquire resistance (R) proteins that recognize these effectors, resulting in a secondary immune response called effector-triggered immunity (ETI) (Pieterse et al., 2009). Global sRNA profiling for specific pest or pathogen interactions have provided useful information regarding the sRNAs involved in immunity and the altered expression of genes, and sRNAs have become the molecular signatures of specific PTI or ETI events. Such molecular markers have been reported for several pathogens, including markers for bacterial, fungal, and viral infections in different plant species (Navarro et al., 2006; Jagadeeswaran et al., 2009; Li et al., 2010; Campo et al., 2013; Feng et al., 2013; Pablo Peláez and Sanchez, 2013). Similar events have been reported during insect herbivory, where several sRNA-regulated defense responses have been identified during herbivory by nematodes and chewing insects (Pandey et al., 2008; Li et al., 2012). Plants infested by phloem-feeding insects belonging to the order hemiptera appear to elicit significantly different responses than chewing insects and might be more closely aligned with responses to biotrophic pathogens. Unlike the chewing pests, sucking insects do not cause massive mechanical wounding to the plant tissue during herbivory. The specialized mouthparts of hemipterans, called stylets, penetrate the cortical tissues to reach the vascular tissues, causing minimal mechanical damage, and evading many of the specialized host defense responses to wounding. However, plants respond to phloem-feeding insects by activating a suite of specific defense responses that are also regulated by sRNAs. This review will primarily focus on the sRNAs involved in plant-hemipteran interactions and will emphasize the role of both plant and insect derived sRNAs in susceptible and resistant host interactions to inform strategies using sRNAs as tools for pest management in agriculture.

### sRNAs in plants

Plants have two major classes of small endogenous RNAs, microRNA (miRNA) and small interfering RNA (siRNA) that are distinguished by their structure and biogenesis. MicroRNAs are derived from single-stranded long primary transcripts (pri-miRNA) that are primarily processed by Dicer-like-1 (DCL1) to a double-stranded hairpin structure called pre-miRNA (Jones-Rhoades et al., 2006; Voinnet, 2009). The pre-miRNA is further processed into the miRNA/miRNA^*^ duplex, which is then methylated by Hua Enhancer 1(HEN1) and loaded into the Argonaute-1 (AGO1)-containing RNA induced silencing effector complex (RISC) (Zhu, 2008; Chen, 2009). Mature miRNA guides RISC to the target mRNA resulting in cleavage and post-transcriptional regulation of the target gene (Mallory and Vaucheret, 2010). In Arabidopsis, miRNAs have also been shown to inhibit the translation of target mRNAs (Li S. et al., 2013). In contrast, siRNAs are derived from double-stranded RNA (dsRNA) precursors that are processed by DCL3 or DCL4 and then loaded in AGO1, AGO7, AGO4, and other AGO complexes (Jones-Rhoades et al., 2006). Other notable characteristics differentiate these two classes of sRNAs. MicroRNAs typically originate from intergenic regions and target unrelated gene loci. In contrast, siRNAs target either the gene from which they are derived or closely related genes. Furthermore, miRNAs are often conserved across closely related species, whereas endogenous siRNAs are highly divergent (Jones-Rhoades et al., 2006).

Small interfering RNAs can be further classified into heterochromatic siRNAs, secondary siRNAs, and NAT-siRNAs (Vaucheret, 2006; Axtell, 2013). Heterochromatic siRNAs are usually 23–24 nt in length and originate from the repetitive and intergenic regions in the chromosome. They are processed by DCL3 and recruit AGO4 as part of the RNAi-induced transcriptional silencing complex and take part in silencing chromatin (Jones-Rhoades et al., 2006; Axtell, 2013). Secondary siRNAs are generated as a “secondary effect” of miRNA-mediated target cleavage. Sometimes the miRNA-mediated cleaved target is used by RNA-dependent RNA polymerase (RDR) to produce secondary siRNAs (Allen et al., 2005; Manavella et al., 2012). This can either give rise to a phased set of siRNAs or trans-acting siRNAs (tasiRNAs) that have the ability to target genes that are different from their loci of origin. Natural-antisense transcript siRNAs (NAT-siRNAs) are generated from dsRNA precursors as a result of hybridization of independently transcribed complementary RNA strands (Borsani et al., 2005; Vaucheret, 2006; Axtell, 2013). These can be further distinguished as *cis*-NAT-siRNA generated from precursors that are transcribed from overlapping regions of the same gene but in opposite polarity, and *trans*-NAT-siRNA whose dsRNA precursors are transcribed from non-overlapping regions, but are complementarity to each other (Borsani et al., 2005; Vaucheret, 2006). There are other classes of siRNAs such as repeat-associated siRNAs (rasiRNA) that have been studied in detail in the maize genome (Barber et al., 2012) and are essential for transcriptional gene silencing and maintaining DNA methylation (Chan et al., 2004; Onodera et al., 2005; Chellappan et al., 2010). The two most recent additions to the repertoire of plant sRNAs are the 21-nt epigenetically activated small interfering RNAs (easiRNA) and siRNAs independent of DCLs (sidRNAs) (Creasey et al., 2014; Ye et al., 2016).

Of all the sRNAs, the miRNAs are the best characterized with well-defined roles in plant development, metabolism, reproduction, defense, and stress biology (Katiyar-Agarwal and Jin, 2010; Sunkar, 2010; Khraiwesh et al., 2012). MicroRNAs can also be classified into two categories: the lineage specific miRNAs found in a single species or across closely related species and the long miRNAs of 23–24 nt in length that are functionally similar to heterochromatic siRNAs (Axtell, 2013).

### sRNAs in insects

Insect sRNAs can be classified into three classes: miRNAs, endogenous-siRNAs (endo-siRNAs), and piwi-interacting RNAs (piRNAs) (Golden et al., 2008). The classification is based on their distinct characteristics, biogenesis, and association with AGO proteins (Kim et al., 2009). Like their plant counterparts, insect miRNAs are well characterized; however, the biogenesis of insect miRNA involves the enzymatic action of two RNase III proteins, Drosha and Dicer. The pri-miRNA hairpin-structure originates from the intergenic region by the polymerase activity of RNA polII and is processed within the nucleus into ~70-nt pre-miRNA by Drosha. The pre-miRNA hairpin lacking perfect complementarity is exported in to the cytoplasm by Exportin-5 where it is processed by Dicer-1 into the miRNA/miRNA* duplex (Lucas and Raikhel, 2013). The 21-nt endo-siRNAs in insects and mammals are produced in an RNA-dependent RNA polymerase (RdRP) independent manner, requiring a Dicer-2-dependent process (Kim et al., 2009). Endo-siRNAs primarily originate from perfect or near complementary regions of transposon transcripts, intergenic repetitive elements, or endo-siRNA cluster loci (Tomari and Zamore, 2005). Piwi-interacting RNAs also originate from intergenic repetitive elements, including retro-transposons, but do not require Dicer for processing. Piwi-interacting RNAs were originally reported from Drosophila germ cells (Lin and Spradling, 1997). Other than their distinct biogenesis, these three classes of sRNA can be distinguished by their size; miRNAs are typically 22 nt, endo-siRNAs are 21 nt, and piRNAs are 24–30 nt (Golden et al., 2008).

Another important characteristic distinguishing the three species of insect sRNAs is their association with distinct members of the Argonaute family. In Drosophila, endo-siRNAs typically use the effector protein Ago-2, an association that is considered to be a distinguishing feature for this class of sRNAs (Golden et al., 2008). Ago-1 acts as the effector protein for miRNAs and in association with GW182 protein, forms the miRISC complex in Drosophila (Tomari et al., 2007; Carthew and Sontheimer, 2009). As their name indicates, piRNAs interact with Piwi proteins. Piwi-interacting RNAs are primarily involved in silencing selfish genetic elements and contribute to germ line stability (Aravin et al., 2007; Hartig et al., 2007). The final distinguishing mark between these three classes is the presence or absence of a 2′-o-methyl modification at the 3′end; siRNAs and piRNAs are modified, whereas miRNAs lack this modification and are therefore susceptible to perioxidate oxidation and beta-elimination (Golden et al., 2008).

### sRNAs in regulating plant interactions with insect pests and pathogens

Plants have developed various defensive strategies to disarm attacks by different insect pests and pathogens. In the last decade, an active role for sRNAs during these plant biotic interactions has been increasingly recognized. Plant-derived sRNAs participate in PTI as well as ETI as defense mechanisms against insect pests and pathogens. However, virulence and host immunity can be affected by pathogen-derived sRNAs that function as effector molecules to overcome the plant immune response (Weiberg et al., 2014). Evidence for the role of miRNAs in PTI was provided by Navarro et al. (2006) when they demonstrated that overexpressing miR393 in Arabidopsis provided enhanced resistance to the bacterial pathogen *Psuedomonas syringae*. Arabidopsis miR393 decreased the steady-state levels of mRNAs encoding auxin receptors transport inhibitor response 1 (TIR1) and auxin signaling F-box 2 and 3 (AFB2, and AFB3), disrupting auxin signaling. As a consequence, auxin-mediated suppression of salicylic acid (SA) is inhibited, impacting plant defense through accumulation of SA and activation of SA signaling. Additionally, in miR393 overexpressing plants, the secondary metabolic pathway is re-directed away from camelaxin toward glucosinolates. The combined effects of enhanced SA signaling and increased levels of glucosinolates contributed to *P. syringae* resistance (Robert-Seilaniantz et al., 2011). Other miRNAs that impact auxin signaling also have been implicated in regulating bacterial pathogenesis. Altered expression of miR160 and miR167 during bacterial infection was linked to differential regulation of the auxin signaling pathway by targeting members of the auxin-response factor (ARF) family of transcription factors (Fahlgren et al., 2007). Plant-derived miRNAs have been implicated in other biotic interactions involving fungi (Lu et al., 2007) and viruses (He et al., 2008). A diverse set of miRNAs was reported to be affected by powdery mildew infection in wheat (Xin et al., 2010). Similarly, Gonzalez-Ibeas et al. (2011) identified a large number of conserved miRNA families in the melon sRNA transcriptome analyzed from watermelon mosaic virus (WMV) and melon necrotic spot virus (MNSV) susceptible (Tendril) and resistant (T-111, and TGR-1551) cultivars. Wheat miR408 negatively regulates plantacyanin TaCLP1, which is responsible for enhanced susceptibility to wheat stripe rust fungus (Feng et al., 2013). Evidence for miRNA-mediated PTI in basal defense against rice blast fungus, has been reported for rice miR169a, miR172a, and miR398b (Li Y. et al., 2014). An exhaustive list of the miRNA families that are involved in bacterial and fungal pathogenesis in several plant species is documented in recent reviews by Weiberg et al. (2014) and Huang et al. (2016).

Specific, and perhaps unique, roles for plant sRNAs have been identified during nematode infection and insect herbivory. Altering global sRNA biogenesis in *dcl* and *rdr* mutants of Arabidopsis showed reduced susceptibility to nematodes (Hewezi et al., 2008), whereas silencing *rdr1* in *Nicotiana attenuata* increased the susceptibility of the plant to herbivory by chewing pests (Pandey et al., 2008). The *rdr1*-silenced *Nicotiana* plants had attenuated expression of jasmonic acid (JA) and ethylene (ET) biosynthetic genes as well as reduced accumulation of JA indicate that sRNAs negatively impact host-defense signaling in response to *Manduca sexta* feeding (Pandey et al., 2008). Additionally, Rasmann et al. (2012) have shown that Arabidopsis mutants deficient in sRNA biogenesis do not inherit the trans-generational priming of jasmonic acid (JA)-dependent defense response against chewing herbivores. Chewing insect herbivory results in significant wound damage to the plant tissues, and several conserved and novel miRNA families a large number of loci generating phased siRNA and tasi-RNA were identified in tobacco in response to herbivory (Tang et al., 2012).

Biotic stress induced by insect pests and pathogens can trigger *R* gene mediated defense responses in plants. Evidence for sRNA regulation of *R* genes in several plant species has increased our understanding of the molecular switch that controls *R* gene mediated responses in plants. During normal plant growth, *R*-gene expression could trigger autoimmunity redirecting the plant metabolism from growth to defense. In the Solanaceae, miRNAs and secondary siRNA have conserved roles in regulating NBS-LRR receptors and innate immunity (Li et al., 2012). For example, NBS-LRR resistance gene mRNAs are specifically targeted by miR482/2118 in tomato and other members of the Solanaceae (Shivaprasad et al., 2012). Similar results were observed in Medicago and soybean where three 22 nt miRNAs (miR1507, miR2118, and miR21090) generated phased-siRNAs that regulate NBS-LRR genes (Zhai et al., 2011).

## Host sRNA pathway components and induced responses against hemipteran herbivory

Phloem-feeding insects belonging to the order hemiptera have adopted a unique feeding niche that exploits the sugar-rich plant phloem sap as a primary food source. Phloem sap is under high turgor pressure that is maintained by low osmotic potentials within transport phloem sieve elements (Taiz and Zeiger, 2010). This sugar-rich environment also contains proteins, peptides, and a high ratio of non-essential: essential amino acids. Phloem-feeding hemipterans have co-evolved to exploit this challenging diet by acquiring several unique adaptions. Phloem feeders have specialized mouth parts, called stylets, which mechanically and enzymatically penetrate cortical cell layers to tap into the sieve element. The high turgor pressure in the punctured sieve element allows sustained passive feeding from the phloem. The osmotic challenges presented by the ingested phloem sap are managed by gut sucrose-transglucosidases that transform excess sugar into long-chain oligosaccharides that is expelled as honeydew (Douglas, 2006). Another unique adaptation is the vertical transfer of symbiotic microorganisms within the gut tissues, providing the insects with essential amino acids that are nutritionally unavailable from the phloem sap diet (Baumann et al., 1997; Douglas, 2006).

Plants are well equipped to protect themselves from phloem feeders. The phloem sap not only provides food, but also has the ability to provide defense against these hemipteran pests (Hagel et al., 2011). The phloem tissue contains secondary metabolites and other defensive compounds that can deter phloem feeders and microbial pathogens. Glucosinolates are sulfur-rich compounds confined in the vacuole of specialized S-cells located in the periphery of phloem tissue of brassicas. During tissue damage, myrosinases, and thioglucosidases present in the M-cells of the phloem parenchyma mix with these glucosinolates to produce toxic isothiocyanates, nitriles, or thiocyanates (Hagel et al., 2011). However, phloem feeders most often evade these defenses by careful stylet insertion during feeding (Tjallingii and Hogen Esch, 1993). Structural phloem proteins also contribute to defense through physical interactions within sieve elements that possibly impact hemipteran feeding. This phenomenon has been best characterized in members of Fabaceae, where spindle-shaped forisomes regulate sieve element occlusion by expanding to spherical structures at sieve plates that occlude the sieve element (Knoblauch et al., 2001; Knoblauch and Peters, 2004; Tuteja et al., 2010). The reversible crystalline to amorphous structural change is determined by calcium flux within sieve elements. Perception of a stress signal activates calcium influx into the phloem sap, resulting in sieve element occlusion. Interestingly, it appears that aphids have salivary calcium chelators that could prevent forisome structural transitions by scavenging calcium within the phloem sap (Will et al., 2007). Emerging evidence suggests that proteases in aphid saliva degrade the very abundant phloem protein 1 (PP1), suppressing a putative phloem defense and providing an additional nitrogen source for the aphids (Furch et al., 2015).

Defense responses against phloem feeders are almost certainly not limited to vascular tissues. While stylet probing is primarily intercellular through the middle lamella of cortical cell walls, intracellular stylet penetration of cells of the cortical tissues is common. This is clearly illustrated by the large number of hemipteran-transmitted viruses that are not phloem-limited and unequivocally confirmed by countless EPG analyses. One weakness in understanding defenses against phloem-feeding insects at the molecular level has been an overall lack of high resolution localization data. Many studies have shown that hemipteran herbivory induces global transcriptional reprogramming in plant tissues that shifts primary metabolism to secondary metabolism and defense (Giordanengo et al., 2010). Defense pathways and related phytohormone-mediated responses are strongly induced in response to hemipteran feeding (Moran and Thompson, 2001; Smith and Boyko, 2007; Morkunas et al., 2011). During the last decade, studies have revealed that sRNAs serve as important modulators of plant stress responses in response to phloem-feeding insects (Greyling, 2012; Sattar et al., 2012b; Barah et al., 2013; Kettles et al., 2013; Xia et al., 2015) (Table 1). Important milestones in our understanding of sRNA function in basal immunity against hemipteran insects have been made in Arabidopsis; however, parallel investigations in non-model systems are revealing the role of sRNAs in host plant resistance. Both approaches are contributing to the future development of integrated pest management strategies.

**Table 1:** **sRNA profiling studies in host plants in response to aphid infestations**.

**Host Plant^a^**	**Insect^b^**	**Interaction**	**Duration**	**Analysis**	**sRNAs Identified**	**References**
Arabidopsis	Cabbage aphid	Susceptible	72 h	miRNA:mRNA co-expression network analysis	Not applicable	Barah et al., 2013
Arabidopsis	Green peach aphid	Susceptible	14 days	sRNA pathway mutant analysis	Not applicable	Kettles et al., 2013
Melon	Cotton-melon aphid	Resistant and susceptible	2–12 h	sRNA sequencing, qRT-PCR	23 conserved miRNA families, 5 novel miRNAs	Sattar et al., 2012a
Chrysanthemum	Chrysanthemum aphid	Resistant	0–48 h	sRNA sequencing	303 conserved miRNAs, 234 novel miRNAs	Xia et al., 2015
Wheat	Russian wheat aphid	Resistant	12–24 h	Subtractive sRNA cloning, qRT-PCR	86 putative miRNAs	Greyling, 2012

a *Melon (Cucumis melo); Chysanthemum (Chysanthemum morifolium); Wheat (Triticum aestivum)*.

b *Cabbage aphid (Brevicoryne brassicae); Green peach aphid (Myzus persicae); Cotton-melon aphid (Aphis gossypii); Chysanthemum aphid (Macrosiphoniella sanbourni); Russian wheat aphid (Duiraphis noxia)*.

### Identifying sRNA co-expression networks and biogenesis pathway components during Arabidopsis-hemipteran interactions

Comparative analyses of the transcriptional changes in Arabidopsis in response to the microbial pathogen *P. syringae* or cabbage aphid (*Brevicoryne brassicae*) revealed commonalities between the two biotic stress signals, as well as aphid-specific responses (Barah et al., 2013). Pathways regulating defense responses, signaling, and metabolic processes were common to both *P. syringae* and the cabbage aphid. Integration of the two data sets by *in silico* analysis of data generated through microarray studies with publicly available gene expression and miRNA datasets for Arabidopsis described a theoretical co-expression network of mRNAs and their cognate miRNAs. The aphid-response network consisted of 82 transcripts, including mRNAs encoding 42 transcription factors and 21 conserved targets for Arabidopsis miRNAs. Further analysis identified 17 miRNA families as regulators of differentially expressed transcripts in response to aphid infestations. Some of these miRNA target transcripts belonged to WRKY and bZIP transcription factor families that have well established functions in biotic stress, reflecting some level of conservation among the different stress responses. The co-expression network also revealed that aphid-specific transcripts were connected to more than one miRNA node, indicating that transcripts are under the regulation of more than one member of a miRNA family or multiple miRNAs belonging to different miRNA families. Additional network complexity was displayed when a single member of a miRNA family was shown to target two different transcripts. While informative, this *in silico* mRNA:miRNA network analysis lacked supporting experimental evidence for miRNA regulation during aphid infestation.

The availability of Arabidopsis mutants for sRNA and defense related pathways provided tools to assess the effects of sRNAs on green peach aphid (*Myzus persicae*) fecundity (Kettles et al., 2013). The reproduction of aphids feeding on *RDR* mutants (*rdr1, rdr2, rdr6*) did not show any differences between these and Col-0 control plants, indicating that interruption of the siRNA pathway had minimal effect on green peach aphid performance in Arabidopsis. Interestingly, *DCL* mutants had differential responses: *dcl1* mutants showed greater resistance toward aphids, but *dcl2, dcl3*, and *dcl4* had no effect on aphid fecundity. Double mutants for *dcl2/3* and *dcl2/4* and triple mutant *dcl2/3/4* also showed no significant change in aphid fecundity. *AGO* mutant *ago1-25* showed significantly reduced aphid fecundity; however, *ago2, ago4*, or *ago7* mutants did not impact aphid performance. Taken together, these data indicate that impaired miRNA processing by specific members of *DCL* and *AGO* multigene families negatively affects reproduction of green peach aphid. This was further confirmed by reduced aphid performance on *hen1, hst* (*hasty*), and *se (serrate)* mutants that also were defective in miRNA processing. Since all the miRNA-processing pathway mutants had a dwarf phenotype, an Arabidopsis line exhibiting a similar phenotype (PDLP1a:GFP overexpression line) was used as a control. It was confirmed that the reduced fecundity was not a result of dwarfism but due to the compromised miRNA processing.

Further analysis of the miRNA-processing mutants revealed that *PAD3* (a marker for camalexin biosynthesis) and *CYP81F2* (member of indolic glucosinolate pathway) (Pfalz et al., 2009) were highly induced at 12 h post aphid infestation in the *dcl1* mutants. HPLC and mass spectrometry analysis confirmed enhanced camalexin content in *dcl1* plants in response to aphid herbivory and it was shown that aphids raised on these mutants ingested camalexin during phloem feeding. Artificial diet assays supplemented with camalexin substantiated the negative impact of this metabolite on aphid fecundity; however, no toxicity was reported for adult aphids. Aphid fecundity assays on *pad3* and *cyp81f* mutants validated the role of camalexin in aphid performance. The impaired miRNA processing pathway also affected phytohormone-mediated defense signaling (Kettles et al., 2013). *LOX2* expression in *dcl1* mutants in response to aphid herbivory was enhanced, whereas, aphid fecundity on *coi1, jar1*, and *35S:LOX2* mutants, defective in JA signaling did not significantly differ from control plants. ET-responsive *HEL* transcript was also induced in response to aphid feeding in *dcl1* plants. Fecundity assays on ET-insensitive *etr-1* or *ein2-5* mutants revealed that aphid reproduction was greater on *ein2* mutant plants, whereas, aphid reproduction on *etr1* mutant plants was not significantly different from control plants. In contrast, previous studies have shown that aphid saliva-induced plant defenses in Arabidopsis did not involve EIN2 and ET signaling (De Vos and Jander, 2009). Thus, EIN2 appears to have some role in green peach aphid resistance that can be seen in either *dcl1* mutants or in the presence of the bacterial effector harpin protein (Liu et al., 2011; Kettles et al., 2013).

### sRNA-mediated resistance against hemipteran insect pests in non-model host plants

Changes in the miRNA profile in response to aphid herbivory have been reported in the ornamental species *Chysanthemum morifolium* showing resistance to chrysanthemum aphid (*Macrosiphoniella sanbourni*) infestations (Xia et al., 2015). Three sRNA libraries were generated from no treatment control plants, plants receiving mock punctures, and aphid-infested plants, respectively. Eighty miRNAs were differentially regulated when comparing the control and aphid-infested libraries; among these 39 miRNAs showed increased expression and 41 miRNAs were down-regulated during aphid herbivory. Comparisons between mock punctures (wounding) and aphid infestation libraries revealed 79 differentially regulated miRNAs, with 39 miRNAs up-regulated and 40 miRNAs down-regulated. Novel miRNAs were also identified from these libraries. Further analysis revealed 24 conserved miRNAs and 37 novel miRNAs were specific to aphid infestations, while of 52 conserved and 9 novel miRNAs were associated with mock puncture (wounding) treatment. In the absence of chrysanthemum genome, the transcriptome was used for *in silico* miRNA target prediction; however, several of the *in silico*-predicted targets could not be verified by experimental methods due to poor coverage of the transcriptome. Because of the lack of validated miRNA targets for chrysanthemum, specific roles for miRNAs in aphid-induced plant defense signaling in the resistant cultivar could not be further explored.

Resistance toward Russian wheat aphid (RWA, *Diuraphis noxia*) is due to the presence of *Dn* genes. Eleven *Dn* genes have been reported from cereals, including *Dn1-9, Dnx*, and *Dny* (Botha et al., 2005). The wheat cultivar TugelaDN contains the *Dn1 R*-gene that confers resistance against RWA biotype 1 (Jankielsohn, 2011). Matsioloko and Botha (2003) observed significant transcriptional changes in response to RWA infestation in the resistant TugelaDN wheat. Genes related to the defense response including receptor and signaling pathway were reported to be differentially regulated within 1–2 h of RWA feeding (Gill et al., 2004; Botha et al., 2005). Subtractive sRNA libraries were constructed from RWA-infested susceptible (Tugela) and resistant (TugelaDN) wheat leaf tissues collected at 12, 18, and 24 h post feeding. The *Dn*-resistance specific sRNAs included 86 putative miRNAs with targets predicted by *in silico* methods (Greyling, 2012). Q-PCR analysis for three selected miRNAs (TaDn-miR65, TaDn-miR15, and TaDn-miR104) showed enhanced expression of these miRNAs in the resistant cultivar in response to aphid feeding in time-course study. Putative targets were predicted for these miRNAs: β*-1, 3 glucanase*, and *cytochrome-P450* targeted by TaDn-miR15 and *WRKY13* and *MYB* targeted by TaDn-65. This demonstrated the potential role for TaDn-miRNAs in aphid resistance.

*R* gene-mediated resistance conferred by the *Vat* (virus aphid transmission) gene against cotton-melon aphids (*Aphis gossypii*) and cotton-melon aphid-transmitted viruses is well documented in melon (*Cucumis melo*) (Kennedy et al., 1978; Dogimont et al., 2014). Resistance to cotton-melon aphids is exhibited as antixenosis (non-preference), antibiosis (delayed growth and development and reduced reproduction), and host plant tolerance (Bohn et al., 1972). The melon miRNA expression profile was determined using sRNAseq combined with comparative analysis of miRNA expression patterns in response to aphid herbivory during resistant and susceptible interactions (Sattar et al., 2012b). Libraries generated from leaf tissues of *Vat*^+^ aphid-resistant melon plants with and without aphids compared the sRNA expression at initial stages of the interaction to distinguish between the molecular cues that are associated with early (2, 4, and 6 h) and late (8, 10, and 12 h) stages that corresponded with pre- and post-sustained phloem ingestion, respectively (Klingler et al., 2001). In total, 23 families of conserved plant miRNAs were identified from the three libraries. Next generation sequence profiling, qPCR, and sRNA blot data revealed that members of 18 conserved miRNA families preferentially accumulated during the early stages of aphid herbivory in the resistant interaction. Twenty-two conserved miRNAs were down-regulated, whereas only one was up-regulated in the early response to aphid infestations. Eight miRNAs were up-regulated during the late stages of aphid herbivory in the *Vat*^−^ susceptible melon. Five miRNA families showed statistically significant down-regulation during early stages and two during the late stages of aphid infestation in the susceptible interaction. Overall, the resistant interaction showed enhanced miRNA expression, whereas the susceptible interaction showed down-regulation of miRNAs. The opposing trends in these nearly-isogenic lines could be due to differences in miRNA transcription or biogenesis. Eighteen cucurbit-specific miRNAs were also identified, five of which were melon-specific, while the remaining 13 sequences were identified from both melon and pumpkin. The expression profiles of all five novel melon-specific miRNAs in *Vat*^+^ resistant melon line did not change significantly during early and late stages of aphid herbivory, but in the *Vat*^−^ susceptible line three were significantly down-regulated during early stages of aphid infestation.

Melon miRNA targets were empirically identified by degradome sequencing and further verified by 5′RNA ligase-mediated rapid amplification of cDNA ends (RLM-RACE) (Sattar et al., 2012b, 2016). Degradome sequencing identified 70 miRNA: mRNA target pairs for the 23 conserved miRNA families that included 28 novel target pairs not found in other plant species. Interestingly, 11 miRNA target gene transcripts encode proteins with established roles in regulating phytohormone (auxin, JA, ET, ABA, and GA) biosynthesis and signaling pathways. A detailed analysis of the miRNA:mRNA interactome revealed six miRNA:mRNA target pairs that impact auxin perception and signal transduction. The auxin-miRNA interactome provided evidence for a series of redundant mechanisms resulting in auxin insensitivity that appears to be a component of *Vat*-mediated resistance (Sattar et al., 2016). Aphid feeding on *Vat*^+^ resistant melon tissues results in miR393-mediated loss of *TIR-1* and *AFB2* auxin receptors. Loss of auxin receptors prevents the formation of SCF^receptor^-ubiquitin ligase complex and degradation of AUX/IAA proteins via the complex. AUX/IAA proteins negatively regulate auxin signaling by inactivating a class of ARF that are transcriptional activators of auxin-induced genes. Simultaneously, miR167 targets ARF activators (*ARF6* and *ARF8*) as a redundant mechanism contributing to auxin insensitivity in the resistant *Vat*^+^ tissue (Sattar et al., 2016). Reduced expression of auxin downstream signaling genes after 12 h of aphid infestation in resistant plants provides indirect evidence for the proposed auxin insensitivity model. Experimental evidence directly linking the inactivation of the auxin receptor with a reduction in aphid fecundity was obtained by treating susceptible melon leaf tissues with a chemical inhibitor (PEO-IAA) of the TIR-1 auxin receptor.

Additional components of the auxin-miRNA interactome in *Vat*-mediated resistance have conserved roles in auxin homeostasis. MicroRNA miR160 targets transcriptional repressor ARF17 that in turn regulates the expression of the gene encoding the *GH3* auxin-conjugating enzyme. MicroRNAs miR164 and miR319 are involved in auxin feedback loops through *NAC* and *TCP* transcription factor genes, respectively, and miR390 mediates miRNA cleavage that generate secondary tasiRNA that target *ARF2* and *ARF3*.

## Insect-derived sRNAs and their role in herbivory

The advent of new sequencing technologies has made it possible for sRNA profiling in hemipteran insect species that have either extensive or limited genomic information. Experimental and *in silico* sRNA profiling studies have been reported for the following phloem feeding insects: pea aphid (*Acyrthosiphon pisum*), cotton-melon aphid (*A. gossypii*), whitefly (*Bemisia tabaci*), brown planthopper (*Nilaparvata lugens*), small brown planthopper (*Laodelphax striatellus*), and white-backed planthopper (*Sogatella furcifera*) (Table 2). Small RNA profiling was reported from the xylem sap feeder glassy-winged sharpshooter (*Homalodisca vitripennis*) and both xylem and phloem feeders Asian citrus psyllid (*Diaphorina citri*) and large milkweed bug (*Oncopeltus fasciatus*) (Table 2). To date, sRNA studies in hemipteran species have primarily focused on identifying sRNA sequences and categorizing those sequences as miRNA, piRNAs, or virus-derived siRNAs (viRNAs). Other studies have identified sRNA biogenesis pathways and sRNAs that are specific to developmental stages, growth, reproduction, or insect immunity. These reports are beginning to provide evidence for sRNA regulation of important biological processes in hemipteran insects and an understanding of insect-host plant and vector-pathogen relationships.

**Table 2:** **Hemipteran sRNAs identified**.

**Hemipteran pest^a^**	**Experimental design**	**Analysis**	**sRNAs identified**	**References**
Whitefly	Comparative analysis of sRNA profile Q and B biotype raised on susceptible host cotton	sRNA sequencing	170 conserved miRNAs and 15 novel candidates	Guo et al., 2014
	miRNA profiles for viruliferous and nonviruliferous whiteflies on tomato	sRNA sequencing, qPCR	112 and 136 conserved miRNAs from nonviruliferous and viruliferous whiteflies	Wang et al., 2016
Glassy- winged sharpshooter	miRNA profiling of insects raised on basil, cotton and cowpea	sRNA sequencing	345 conserved and 14 novel miRNAs	Nandety et al., 2015
Pea aphid	*In silico* prediction of miRNAs from genome sequence	Solexa sequencing	149 miRNAs including 55 conserved and 94 new miRNAs	Legeai et al., 2010
	miRNA and siRNA pathway identification	Annotation of the miRNA and siRNA pathway genes and expression profiling of these genes	Not applicable	Jaubert-Possamai et al., 2010
	Evolutionary analysis of the miRNA machinery	Phylogenetic analysis of *ago-1* and *dcl-1*	Not applicable	Ortiz-Rivas et al., 2012
Cotton- melon aphid	Comparative analysis of insects feeding on susceptible and resistant melons	sRNA sequencing	81 conserved miRNAs, 12 aphid-specific miRNAs, 9 novel miRNA candidates	Sattar et al., 2012a
	Analysis of ESTs	*In silico*	16 potential miRNAs	Rebijith et al., 2014
Brown planthopper	Prediction of novel miRNA	*In silico*	9 novel miRNAs	Asokan et al., 2013
	Comparative analysis of sRNA from the insect developmental stages	sRNA sequencing	452, 430, and 381 conserved miRNAs from adult male, adult female and female nymph libraries	Chen et al., 2012
	Genome-wide screening for siRNA, miRNA pathway		Not applicable	Xu et al., 2013
	Analysis of fecundity-related miRNAs	Dual-luciferase assay, miRNA injection	38 potential miRNAs regulating 9 fecundity-related genes	Fu et al., 2015
	Identification of miRNAs regulating molting	sRNA sequencing, miRNA injections, qRT-PCR	miR-8-5p and miR-2a-3p regulate chitin synthesis	Chen et al., 2013
	Analysis of sRNA biogenesis gene *dcl-1*	Cloning and sequencing of *dcl1*, qRT-PCR of *dcl* in different tissues	Not applicable	Zhang et al., 2013
Small brown planthopper	RBSDV infection	sRNA seq	59 conserved miRNA, 20 novel miRNAs	Li et al., 2015
	HiPV-derived sRNAs	sRNAseq	Virus derived RNAs are 21–22 nt	Li J. et al., 2014
Asian citrus psyllid	Prediction of virulence-regulatory miRNAs and phylogenetic analysis of miRNA clades	*In silico*	10 major clades	Khalfallah et al., 2015
Large milk-weed bug	Prediction of miRNAs	*In silico*	96 candidate mature miRNAs	Ellango et al., 2016
White-backed plant hopper	Small RNA libraries from viruliferous and non-viruliferous insects	sRNA sequencing	106 conserved miRNAs, 276 novel miRNAs	Chang et al., 2016

a *Whitefly (Bemicia tabaci); Glassy-winged sharpshooter (Homalodisca vitripennis); Pea aphid (Acyrthosiphon pisum); Cotton-melon aphid (Aphis gossypii); Brown planthopper (Nilaparvata lugens); Small brown planthopper (Laodelphax striatellus); Asian citrus psyllid (Diaphorina citri); Large milkweed bug (Oncopeltus fasciatus); White-backed planthopper (Sogatella furcifera)*.

### Identification of sRNA pathways in hemipteran insects

The pea aphid has become the model hemipteran species due to an international collaborative effort to obtain the fully sequenced and annotated genome, which has opened avenues for fundamental studies to be conducted in this species. MicroRNA sequences as well as genes involved in siRNA and miRNA biogenesis from pea aphid were initially predicted by *in silico* probing of the genome sequence (Jaubert-Possamai et al., 2010; Legeai et al., 2010; Kozomara and Griffiths-Jones, 2011). Phylogenetic analysis revealed duplicated miRNA biogenesis genes in the pea aphid (two *Ago-1*, two *Dcr-1*, and four *Pasha* gene copies) that retain their functionality (Jaubert-Possamai et al., 2010). These duplication events occurred at different time periods with the *Dcr-1* duplication being a recent event, while *Ago-1* occurred as an ancestral event in the subfamily Aphidinae. The *Ago-1* and *Dcr-1* duplicated genes were differentially expressed in four different reproductive morphs of the pea aphid (Ortiz-Rivas et al., 2012). Duplication events were also reported for genes from the pea aphid piRNA pathway (Lu et al., 2011).

Aphids have unusually high phenotypic plasticity and can switch from sexual to asexual reproduction (Miura et al., 2003), which presents a unique system to investigate the role of duplication events in the piRNA biogenesis pathway during asexual and sexual reproduction. Expression of the duplicated *Piwi* and *Ago* genes was tissue specific in certain reproductive morphs (Lu et al., 2011). During embryogenesis, *Api-Piwi2, Api-Piwi6*, and *Api-Ago-3a* were expressed in germ cells, whereas duplicated copies *Api-Piwi5, Api-Piwi3*, and *Api-Ago3b* were localized in somatic cells. Semi-qPCR detected differential expression for *Api-Piwi* and *Api-Ago3* genes in the different reproductive morphs. *Ago-3b* was most abundant in the sexuparae female morph, whereas *Ago-3a* was abundantly expressed in all of the female morphs. Both the *Ago-3* duplicates were expressed at very low levels in the sexual males, indicating *Ago-3* was not involved in male sexual reproduction. Expression studies of *Api-Piwi* genes in the different reproductive morphs revealed germ line-specific *Api-Piwi2* and somatic cell-specific *Api-Piwi3* were abundant in all the female reproductive morphs. Interestingly another somatic cell-specific *Api-Piwi5* was strongly expressed in the sexual males. *Api-Piwi6* was strongly expressed in the germline cells of the female oviparae. These data indicate additional functions for *Piwi* genes during both sexual and asexual phases of aphid reproduction.

Several genes belonging to the different sRNA pathways were identified from the soybean aphid (*Aphis glycines*) (Bansal and Michel, 2013). Single copies of *Dcr2, R2d2, Ago2*, and *Sid-1* were identified in soybean aphid. Expression analysis of the sRNA pathways genes at different developmental stages showed *Dcr2, R2d2*, and *Ago2* were highest during the first and second instar stage. However, *Sid-1* was uniformly expressed throughout all the developmental stages in the soybean aphid. Tissue-specific qPCR analysis detected the presence of *Dcr2, R2d2, Ago2*, and *Sid-1* in the epidermis, gut, and fat body of the insect. Because *Sid-1* is essential for systemic response of RNAi in both *Apis mellifera* and *Caenorhabditis elegans* (Winston et al., 2002; Aronstein et al., 2006), its presence throughout all the developmental stages opens up the possibility of designing effective RNAi-mediated control of the soybean aphid.

Small RNA pathways also have been evaluated and characterized in brown planthoppers (Zha et al., 2011; Xu et al., 2013). Brown planthopper *Sid-1* and *Aub* genes encoding proteins involved in the RNAi pathway were identified, as were members of the *Ago* and *Dcr* families (Zha et al., 2011). Genome and transcriptome sequence analyses revealed one *Drosha*, three *Dcr* genes, and one ortholog each of the RNA-binding protein *R2D2, Loquacious* (*Loqs*), and *Pasha* (Xu et al., 2013). Three members of the *Ago* family (*Ago-1, Ago-2*, and *Ago-3*), were also identified, indicating the presence of siRNA, miRNA, and piRNA pathways in the brown planthopper (Xu et al., 2013). The brown planthopper sRNA pathway genes were cloned, sequenced, and their functionality confirmed by gene knockdown assays using dsRNA microinjections. The brown planthopper nymphs with *Sid-1* knockdown lost systemic RNAi for other targets, confirming the conserved role for *Sid-1* in this insect. Third-instar brown planthopper nymphs with silenced *Dcr-1* and *Ago-1* showed lethal defects, and the few that survived could not complete metamorphosis nor were able to stretch their wings (Xu et al., 2013). These experiments suggest that miRNA pathways impact insect development and ecdysis. Zhang et al. (2013) observed a similar effect for *Dcr-1* down-regulation in brown planthopper adult females. Microinjection of *Dcr1* into adult females caused significant loss of *Dcr-1* transcripts in both whole body and ovaries. Furthermore, the oocytes of the adults with *Dcr-1* knockdown were poorly developed with abnormal follicular development. As a result the number of eggs produced by *Dcr-1*-silenced brown planthopper females where significantly less than those in the control group. Also, the expression of several ubiquitously found conserved miRNAs (*bantam, miR-7, miR-8*, and *miR-9*) decreased significantly in ds*Dcr1*-treated brown planthopper adult females 3 days following microinjection.

Zhou et al. (2016) demonstrated the differential expression of *Ago-1* and *Ago-2* in small brown planthoppers under different stress conditions. Although both *Ago* genes are expressed during all developmental stages of the insect, reduced expression of both *Ago-1* and *Ago-2* was reported in second-instar small brown planthopper nymphs in response to rice black-streaked dwarf virus. Both high and low temperature extremes negatively affected *Ago-1* expression; however, *Ago-2* expression was markedly reduced only in response to low temperature stress. Changing host plants initially caused reduced expression of both the *Ago* genes, but the expression of *Ago* genes recovered to their normal state after a 7-day period on the new host, indicating that *Ago* genes have important roles host specificity as well as stress responses. Other important genes from the RNAi pathway such as *Eri-1* and *Sid-1* were also identified from the small brown planthoppers.

### sRNAs regulating insect development, growth, and reproduction

Hemipterans are paurometabolous insects with three life stages (egg, nymph, and adult) that undergo gradual metamorphosis (Bybee et al., 2015). For example, aphid nymphs molt 6–8 times and then metamorphose into an adult. Reproduction in hemipterans can be sexual or asexual. Some hemipterans, such as aphids, are economically important agricultural pests with prolific reproductive ability. When favorable conditions exist, aphids reproduce asexually, giving birth to live females rather than laying eggs. As days shorten and become cooler, aphids produce winged males and females that can mate and reproduce sexually to overwinter as eggs on perennial host plants (Ogawa and Miura, 2014). Female aphids begin reproducing parthenogenetically 7–10 days after birth. The reduced pre-reproductive period is possible because of “telescoping of generations” where aphids complete much of their development, including their reproductive system before they are born (Dixon, 1998). Aphid growth and development are reliable indicators of insect performance on host plants because they correlate with fecundity and are directly impacted by environmental factors (Awmack and Leather, 2007).

Insect growth, development, reproductive potential, and interactions with plant hosts can be influenced by sRNAs (Asgari, 2013; Lucas and Raikhel, 2013). In Drosophila, miRNAs have been identified as regulators of reproductive biology, including differentiation and maintenance of germlines within the ovaries (Park et al., 2007). Genome-wide association studies have identified several Drosophila miRNAs as well as epigenetic modifications associated with sexual reproduction and ageing (Zhou G. et al., 2014; Zhou S. et al., 2014). The potential role sRNAs on pea aphid reproduction and life cycle was first suggested by Ortiz-Rivas et al. (2012) when they reported differential expression of *Ago-1* and *Dcr-1* genes in the asexual and sexual reproductive morphs. As the aphid lifecycle transitions from asexual to sexual reproduction, the sexupara females parthenogenetically produce sexual morphs and the females carrying eggs mate with the male. PCR-based expression assays confirmed *Ago-1a* and *Dcr-1b* overexpression in sexupara females. The *Ago-1a* was down-regulated in sexual female morphs, whereas *Ago-1b* was down-regulated in asexual females reproducing parthenogenetically, and *Dcr-1b* was not expressed in the sexual males. These observations indicate specific functions for the duplicated gene copies of *Ago-1* and *Dcr-1* during the reproductive transition in pea aphid (Ortiz-Rivas et al., 2012).

Differential expression of sRNAs across different reproductive morphs was also observed in other hemipteran insects. Comparative analyses of the sRNA libraries from different developmental stages of the brown planthopper were conducted to identify sRNAs associated with insect growth and development (Chen et al., 2012). A bimodal distribution pattern of sRNAs were observed for the three libraries: 21–22 nt sRNAs were predominant in adult males; 26–27 nt sRNAs were abundant in adult females; and an almost equal distribution of 22-nt and 28-nt sRNAs in the last instar of female nymphs. Analysis of a subset of the conserved miRNAs revealed that miR30d was specific to female adults and nymphs, whereas miR-144^*^ and miR-20d were exclusively expressed in female nymphs. Certain miRNAs (miR-1, miR-184, miR-278, and miR-34) were highly expressed in adult males. The conserved miRNAs bantam and miR-10 were ubiquitously present in all three reproductive morphs. Novel miRNAs identified from brown planthoppers also showed differential expression within the reproduction morphs. MicroRNA bph-m0032 was exclusively expressed in female adults, whereas bph-m0045 was only found in female nymphs, and two novel miRNAs bph-m0057 and bph-m0041 were found in both male and female adults.

Additional studies of sRNAs in the brown planthopper identified two conserved miRNAs miR-8-5p and miR-2a-3p that modulate the chitin biosynthetic pathway membrane-bound trehalase (*Tre-2*) and phosphoacetylglucosamine mutase (*PAGM*), respectively (Chen et al., 2013). Both miR-8-5p and miR-2a-3p were highly expressed in nymphs and both female and male adults. During molting, miR-8-5p and miR-2a-3p and their respective target genes *Tre-2* and *PAGM* showed anti-correlated expression patterns with the enhanced expression of both miRNAs and down-regulation of the respective targets on the last day of 3rd, 4th, and 5th instars. The differential expression of miR-8-5p and miR-2a-3p and their respective targets between the first day of a new instar and last day of previous instar suggests a strong correlation to changes induced by the steroid hormone 20-hydroxyecdysone (20E) during the molting process. Co-transfection of miR-8-5p and miR-2a-3p along with the respective targets fused to a luciferase reporter gene in the human embryonic kidney cell line HEK293T and Drosophila derived S2 cell lines showed decreased expression in dual luciferase assays. Microinjection experiments with synthetic dsRNA copies of endogenous miRNAs (miRNA mimic) in the 5th instar confirmed the dual luciferase assay results and showed reduced expression of the target proteins. Nymphs feeding on an artificial diet containing the miR-8-5p mimic experienced starvation-related mortality, while those fed a diet containing the miR-2a-3p mimic showed severe molting defects. Diet assays with miRNA inhibitors had no adverse effect on brown planthopper nymphs. Chitin content in these nymphs was significantly reduced in those fed with miRNA mimics, whereas the nymphs from the inhibitor assay had enhanced chitin content as compared to the control group. Furthermore, experimental evidence showed that both miR-8-5p and miR-2a-3p were negatively regulated by ecdysone-inducible gene *BR-C* by 20E signaling during brown planthopper molting. This study directly links miRNAs to chitin biosynthesis during insect development that is regulated by the steroid hormone 20E.

Insect fecundity is an important trait to predict population growth rates on host plants and forecast their performance under field conditions (Awmack and Leather, 2007). Fecundity also serves as a reliable measure of the plant host-insect interaction and is especially valuable when screening plant genotypes for resistance. Reduced fecundity is a hallmark of *Vat*-mediated resistance in melon to the cotton-melon aphid (Klingler et al., 1998). In addition to reduced fecundity, aphids on resistant plants have an extended pre-reproductive period and shortened reproductive and post-reproductive periods resulting in fewer progeny. The overall life span of an individual aphid is reduced and after the final molt, aphids feeding on resistant plants are smaller in size than those feeding on the susceptible melon plants (Kennedy and Kishaba, 1977; Klingler et al., 1998). Comparative analysis of sRNA libraries from aphids feeding on *Vat*^+^ and *Vat*^−^ plants for 48 h showed a differential bimodal size distribution pattern for sRNAs in the two libraries with the *Vat*^+^ library over-represented by longer 26–27 nt sequences (Sattar et al., 2012a). Approximately half of these longer sRNA sequences mapped to transposable elements. In insects, a vast majority of the sRNA sequences that arise from the transposable elements are endogenous piRNAs involved in maintaining genome integrity (Biryukova and Ye, 2015). A search of *Buchnera aphidicola* homology revealed 4.6% of the 26–27 nt sequences in the *Vat*^+^ library were of bacterial origin. Although there is no direct experimental evidence implicating the role of endosymbiont-derived sRNAs in aphid reproduction during *Vat*^+^ interactions, previous studies in other aphid species have confirmed that the endosymbiont *B. aphidicola* is required for successful reproduction (Srivastava and Auclair, 1976; Douglas, 1992; Dunbar et al., 2007; Shigenobu and Wilson, 2011). A detailed discussion of endosymbiont-derived sRNAs by Hansen and coworkers is presented in this focus issue. In addition to the longer sequences, a total of 81 miRNAs belonging to 56 miRNA families were identified from cotton-melon aphid libraries (Sattar et al., 2012a). While putative target genes have been predicted by *in silico* methods the role that these miRNAs play in aphid reproduction and their relationship to host plant resistance remains to be determined.

Reduced fecundity was observed for soybean aphids, feeding on bean pod mottle virus (BPMV)-infected host plants (Cassone et al., 2014). BPMV is not vectored by soybean aphids, yet the presence of the virus showed a negative impact on aphid fecundity. Although RNAseq analysis of the aphids did not reveal the presence of transcripts associated with viral immunity, sRNA biogenesis genes belonging to the siRNA, miRNA, and piRNA biogenesis pathways were down-regulated in aphids feeding on BPMV-infected host plants, indicating a defense response. However, viral replication for BPMV was not observed in the soybean aphid and Cassone et al. (2014) speculate that the loss of fecundity may be a result of aphids investing more in “survival rather than reproduction” due to limited resources available in virus-infected plants.

MicroRNAs regulating fecundity were identified in adult brown planthoppers (Fu et al., 2015). MicroRNA-binding regions in the 3′-UTR of fecundity-associated genes detected *in silico* led to the identification of 38 miRNAs targeting nine fecundity genes. Among these 38 putative miRNAs, miR-4868b showed perfect complementarity to the 3′UTR region of the *glutamine synthetase* (*GS*) gene. The miR-4868b:*GS* target pair was confirmed using the dual-luciferase assay reporter assay for the *GS* target in S2 cell lines. Microinjecting newly emerged adult female planthoppers with the miR-4868b mimic reduced GS protein levels within 48 h; however, the accumulation of GS mRNA did not change, indicating miR-4868b regulated the expression of GS protein by translational repression. GS protein also accumulated following treatment with a miR-4868b binding inhibitor. The number of offspring in the miR-4868b-mimic treatment decreased by 32% compared with the control group, illustrating the effect of reduced GS protein on fecundity. Ovaries isolated from adult females 2 days after the miR-4868b mimic treatment showed delayed development, fewer ovarioles, and fewer developed eggs per ovary. Earlier studies using RNAi-mediated knockdown of GS protein in brown planthopper have resulted in severe defects in ovary development and egg deposition (Zhai et al., 2013). Taken together they confirm miR-4868b plays a role in brown planthopper reproduction via regulation of GS. Vitellogenin (*Vg*) was also reduced by the miR-4868b mimic treatment. However, negative effects of microinjecting the miR-4868b mimic on *Vg* expression and ovarian development were transient with no significant differences between the treatment and control groups 6–7 days post-microinjection. The link between *GS* and *Vg* in brown planthopper reproduction is not fully understood, but may be through the glutamine-activated TOR signaling pathway. Several studies have shown TOR signaling pathway plays a role in insect fecundity by regulating *Vg* accumulation and ovary development (Patel et al., 2007; Zhai et al., 2015).

### Hemipteran sRNAs in response to virus infection

Hemipteran insects, especially members of the Aphididae, are common vectors of plant viruses and play significant roles in viral epidemiology. Viruses transmitted by aphids outnumber those transmitted by whiteflies, leafhoppers, and planthoppers combined (Nault, 1997). The majority of aphids transmit “stylet borne” viruses in a non-persistent manner, where a very brief stylet penetration of less than a minute is required for viral acquisition and inoculation of the host plant (Katis et al., 2006). Some aphids, however, transmit viruses in a semi-persistent manner where longer periods are required for acquisition and inoculation of viral particles. Persistent transmission requires a latent period between viral acquisition and viral inoculation allowing the virus to propagate or only circulate within the aphid during the course of its lifetime (Katis et al., 2006).

Antiviral immunity in both plants and insects is mediated by RNA interference (RNAi) (Ding and Vionnet, 2007; Obbard et al., 2009). Virus-derived siRNAs accumulate during viral infection in plants and insects cleaving the viral dsRNA into short fragments causing silencing of the viral genes in a systemic manner (Ding and Vionnet, 2007; Wieczorek and Obrępalska-Stęplowska, 2015). Concurrently, viruses evolved a counter mechanism for viRNA-mediated silencing by producing viral suppressors of silencing (VSR). VSR proteins interfere with RNA silencing by specifically targeting components of the RNA-silencing pathway (Ding, 2010). Members of the RNA silencing (*Dcr-2* and *R2D2*) and piRNA biogenesis pathways have been implicated in insect viral immunity (Zambon et al., 2006; Vodovar and Saleh, 2012). Long viral-derived sRNAs similar to endogenous piRNAs have been reported upon viral infection in Drosophila ovarian somatic sheet cells, although it could not be confirmed if they originated from the piRNA biogenesis pathway (Wu et al., 2010). Understanding the role hemipteran sRNAs play in viral immunity could enable new approaches in preventing the systemic spread of plant viruses.

Researchers have investigated sRNA pathways in several hemipteran species as a response to virus acquisition and infection in host plants (Li et al., 2013a; Sekhar Nandety et al., 2013; Li J. et al., 2014; Li et al., 2015; Chang et al., 2016; Wang et al., 2016). Comparing sRNA sequences from small brown planthoppers infected with rice black-streaked dwarf virus (RBSDV) and rice stripe virus (RSV) revealed the greatest accumulation of viRNAs during RBSDV infection (Li et al., 2013a). RBSDV induced viRNAs were predominantly 21–22 nt in length originating in equal proportions from the sense and antisense strands. Hotspots for viRNA initiation were restricted to the 5′ or 3′ terminal regions of viral genome. Double infection of RBSDV and RSV induced more viRNA from the RBSDV RNA segments. In addition to the RBSDV- and RSV-derived virRNAs, Himetobi P virus (HiPV)-derived viRNAs were identified in the sRNA libraries (Li J. et al., 2014). Subsequently, HiPV infection was confirmed in the insect host. Analysis of all virus-infected and uninfected samples revealed greater accumulation of HiPV-derived RNA in the RSV library than in the RBSD or the double-infection library, suggesting that HiPV abundance is determined by the RSV infection. Although viral infection in insects typically produces *dcr-2* derived 21–23 nt viRNAs, HiPV-derived viRNAs showed a wide range of size distribution from 18 to 30 nt. Majority of the 21–22 nt viRNAs were generated from the antisense strand, whereas the longer viRNAs came from the sense strand. While initially thought to be piRNAs, these long sequences lacked the characteristic piRNA peak at 27–28 nt and uracil bias at the 5′-terminal end. The authors concluded that these long RNAs were likely derived from the sense strand from the viral genome by an unknown sRNA biogenesis pathway.

Differentially expressed miRNAs in response to the virus infection were identified in RBSDV-infected small brown planthoppers (Li et al., 2015). Nine up-regulated and 12 down-regulated conserved miRNAs were identified from the RBSD-infected library. Several miRNAs (miR-2765-5p, miR-87-3p, and miR-1-3p) were induced, while others were repressed (miR-750-3p, miR-727-5p, miR-124-3p, and miR-133-3p) in the insect host. Twenty novel miRNA candidates were also identified in this interaction. Target identification for these miRNAs was hampered by the lack of small brown planthopper genome sequence data. In the future, validated miRNA targets will provide a better understanding of the physiological significance of miRNAs in RBSD infection of small brown planthoppers.

Analysis of sRNA libraries prepared from white-backed planthoppers (*S. furcifera*) infected with southern rice black-streaked dwarf virus (SRBSDV) identified eight up-regulated miRNAs and four down-regulated miRNAs, among which two, miR-14 and miR-2798, are conserved miRNAs and the remaining 10 are unique to the insect (Chang et al., 2016). MicroRNAs miR-14 and the novel miR-n98a target genes involved in viral immunity. The highly expressed miR-14 targets transcripts encoding the patched (Ptc) protein, a positive regulator of hedgehog signaling. The hedgehog signaling pathway has been implicated in host interactions with dengue virus in *Aedes aegypti* (Chauhan et al., 2012). SFU-20.387 mRNA, encoding a Rab-5 interacting protein with a well-established role in Hepatitis C virus genome replication in mammals is the putative target for *S. furcifera* miR-n98a (Stone et al., 2007). Based on these observations, it was speculated that miR-14 and miR-n98a are involved in SRBSDV virus infection and immunity (Chang et al., 2016).

Homologs of sRNA biogenesis genes *ago-1* and *dcr-1* have been identified from whiteflies infected with begomovirus (Wang et al., 2016). sRNA profiling from viruliferous and non-viruliferous whiteflies carrying tomato yellow leaf curl China virus (TYLCCNV) showed an abundance of larger 29–30 nt sRNAs in the non-viruliferous library, whereas the viruliferous library was enriched in smaller 21–22 nt sRNA sequences. The whitefly miRNA profile was also analyzed in response to virus infection. Among the 52 miRNAs that were differentially expressed in the nonviruliferous and viruliferous libraries, 26 were specific to the viruliferous library. The expression of these miRNAs was confirmed by qPCR: miR-bantam, miR-1, miR-2b, and miR-124 were significantly up-regulated and miR-307, miR-317, and miR-993a were down-regulated in the viruliferous library. In addition to conserved miRNAs, seven novel miRNAs were identified from both the libraries. *In silico* predicted target genes of the differentially expressed miRNAs primarily belonged to three main GO categories: biological processes, cellular processes, and molecular function.

The glassy-winged sharpshooter is a xylem-feeding leafhopper that is an important pest on a wide range of plants including citrus, grapes, and almonds and vectors *Xylella fastidiosa*, the causal agent of Pierce's disease of grapevines and citrus-variegated chlorosis disease. Sekhar Nandety et al. (2013) identified viRNAs in glassy- winged sharp shooters infected with either *Homalodisca coagulata virus-1* (HoCV-1) or *H. vitripennis reovirus* (HoVRV) and mapped the viRNAs to the viral genomes. Most of the viRNA sequences for HoCV-1 were derived from the positive strand, whereas HoVRV sequences were evenly distributed across the genome. In contrast to HoCV-1 viRNAs, several hotspots were identified for HoVRV on both 5′ and 3′ ends of the viral segments. The distinct mapping patterns for viRNAs from two taxonomically different viruses in the same insect vector raises the possibility of unique anti-viral immunity targets for each virus.

The combined effect of two taxonomically different viruses on viral immunity in an insect host was documented in soybean aphid (Vijayendran, 2014). A novel viral pathogen, *A. glycines virus* (AGV) was identified from the transcriptome sequencing of the soybean aphid. AGV infection was ubiquitously present in several clonal populations of soybean aphids collected from different geographical locations. The enhanced transfer rate of AGV to different insect hosts is possibly due to its ability to evade the RNAi-mediated anti-viral host defense. AGV is susceptible to RNAi-mediated anti-viral immunity in the host, but only in the presence of another viral pathogen *Aphid lethal paralysis virus* (ALPV). This was clearly demonstrated by a reduction of viRNAs produced from the AGV genome as compared during AGV infection alone. In contrast, a large number of viRNAs were produced in response to the double infection by AGV and ALPV, and the majority of these viRNAs were mapped to the ALPV genome.

## sRNAs as a tool in agriculture for hemipteran pest control

Insects and the microbial pathogens they vector are major causes of economic losses in production agriculture. Developing species-specific and environmentally benign approaches are important considerations when designing pest management strategies. RNA interference (RNAi) technology appears to be a promising candidate for such an approach. During RNAi, dsRNA is cleaved by Dicer to generate 21–24 nt siRNAs. The siRNAs separate into guide and passenger strands; the guide strand is introduced into the RISC and the passenger strand is degraded (Agrawal et al., 2003; Meister, 2013). The discovery of RNAi machinery in economically important hemipteran pests, including pea aphids, soybean aphids, whiteflies, brown planthoppers, and small brown planthoppers provides a robust rationale to pursue RNAi-based pest management strategies for hemipterans (Jaubert-Possamai et al., 2010; Ortiz-Rivas et al., 2012; Bansal and Michel, 2013; Xu et al., 2013; Wang et al., 2016; Zhou et al., 2016). RNAi protocols for hemipterans typically introduce dsRNA into the insect by one of several experimental methods: microinjection where dsRNAs are directly injected into the body of the insect; feeding dsRNAs in artificial diets or *in planta*; direct topical application by spraying or soaking insects in dsRNA solutions; or incorporating dsRNAs into nanoparticles (Scott et al., 2013). The mode of introducing dsRNA into the insect and the tissue in which the target gene is expressed are important criteria to obtain successful gene silencing in hemipteran insects.

### Direct delivery of dsRNA or siRNA via injections in hemiptera

Microinjection has been successfully used to deliver RNAi in several insect species belonging to lepidoptera, coleoptera, diptera, as well as hemiptera (Yu et al., 2013). RNAi-mediated silencing of *Hox, wg*, and *decapentaplegic (dpp)* in large milkweed bug (*O. fasciatus*) (Angelini and Kaufman, 2005) and salivary gland gene *Coo2*, gut-specific *cath-L* genes, and *calreticulin* in pea aphid (Mutti et al., 2006; Jaubert-Possamai et al., 2007) employed microinjection to deliver the dsRNA. Microinjecting brown planthoppers with dsRNA against *calreticulin, cathepsin-B*, and nicotinic acetylcholine receptors (nAChRs) β2 subunit *Nl*β*2* resulted in ~50% silencing effect; however, high insect mortality is often reported especially in smaller insects as a result of wounding during microinjection (Liu et al., 2010; Li et al., 2013b).

### Oral delivery of dsRNA in hemiptera

Oral delivery through diet is a less invasive method for introducing dsRNA into hemipteran insects (Scott et al., 2013). Unlike microinjection, oral delivery of dsRNA through feeding sachets does not result in wounding-induced mortality and can be a useful tool when working with smaller insects. However, it is difficult to quantitate the dsRNA dose ingested by the insects to produce the silencing effect and thus, higher dosages are often required for oral delivery. In the absence of systemic RNAi machinery, the success of oral delivery may be limited to gut-specific target genes. Pea aphids feeding on an artificial diet supplemented with dsRNA against *aquaporin* showed 50% silencing of aquaporin transcript (Shakesby et al., 2009), whereas lethal effects were obtained in response to orally administered dsRNAs against gut *vATPase* (Whyard et al., 2009). Such effects could be species specific, as in the brown planthopper where orally-delivered *vATPase* dsRNA resulted in only ~50% silencing of vATPase subunit E (Li et al., 2011), whereas dsRNA against *trehalose phosphate synthase (TPS)* showed a marked reduction in *TPS* activity in the fat body, ovary, and midgut (Chen et al., 2010). Diet-delivered dsRNA-mediated silencing of *sugar transporter gene 6 (Nlst6)* showed reduced *Nlst6* expression in the midgut with a negative effect on brown planthopper growth and fecundity (Ge et al., 2015). In whitefly, diet-delivered dsRNA against *glutathione S-transferase (GST)* showed significant decreases in mRNA levels that correlated with mortality in the insects (Asokan et al., 2015).

Chaitanya et al. (2016) studied effect of gene silencing using the sachet diet method to deliver dsRNA to cotton-melon aphid. Aphids fed on sachet diets containing dsRNA specific to sodium channel (*AgSCN*) or ultraspiracle genes (*AgUSP*) resulted in high levels of mortality that corresponded to decreased transcript levels for both genes. Oral delivery of dsRNA to silence cotton-melon aphid *juvenile hormone binding protein (JHBP)* and *vacuolar ATPase subunit H (V-ATPase-H)* showed a 10–73% reduction in mRNA for both JHBP and *V-ATPase-H* and mortality in the range of 10–63% for both treatments (Rebijith et al., 2016). Comparative analysis of microinjection and oral delivery of dsRNA targeting the *cathepsin-L* gene in pea aphid demonstrated efficacy for each method that was tissue or organ specific (Sapountzis et al., 2014). Microinjection was most successful for gene knockdown in the head and carcass that induced altered morphology. In contrast, diet delivery showed enhanced silencing effect in the gut and gut-specific epithelial cells, possibly due to weak systemic spread of the RNAi signal. A similar study in potato/tomato psyllid (*Bactericera cockerelli*) compared the efficacy of microinjection and oral feeding (Wuriyanghan et al., 2011). Double-stranded RNAs introduced into the psyllids were experimentally shown to be processed into 21-nt siRNAs. Although microinjections were effective for dsRNA-mediated silencing for *actin*, mortality rates due to wounding were also higher. Sachet feeding of dsRNA or siRNA targeting *actin, ATPase, hsp70*, and *CLIC* showed tissue-specific gene knockdown of actin in the gut tissue, whereas silencing of the other genes was achieved in the whole insect (Wuriyanghan et al., 2011). The gut-specific knockdown of actin could be due to the lack of a systemic RNAi response for potato/tomato psyllid *actin* transcript.

### In planta delivery of dsRNA

Expressing dsRNA within the host plant, either transiently or through stable integration, allows the effects of specific gene targeting on insect performance to be evaluated in the most relevant environment. The effect of *Rack1* and *Coo2* gene silencing on green peach aphid performance and fecundity was evaluated in *Nicotiana benthamiana* and Arabidopsis plant tissues (Pitino et al., 2011). Aphid gut-specific *Rack1* and salivary gland-specific *Coo2* transcripts were down-regulated in aphids feeding on *N. benthmiana* leaves transiently expressing ds*Rack1* and ds*Coo2*. Transient expression experiments reduced aphid fecundity by 25%, whereas, ds*Rack1* and ds*Coo2* transgenic plants showed a 50–60% decrease in mRNA levels with a 20% reduction in aphid fecundity. Neither method negatively affected aphid survival. These results deviated from the earlier microinjection studies in pea aphid where ds*Coo2* was lethal (Mutti et al., 2006).

Guo et al. (2014) compared two distinct approaches to gene silencing by developing *N. benthamiana* transgenic lines carrying intron-spliced hairpin RNA (hpRNA)-expressing plant vectors for *acetylcholinesterase 2 (MpAChE2), vATPase*, and *tubulin folding cofactor D (TBCD)* or artificial miRNAs (amiRNAs) targeting two different sites in the Mp-*AChE2*. Transgenic tobacco plants expressing Mp-*vATPase* and Mp*-TBCD* hpRNAs showed enhanced resistance toward green peach aphids with ~30% reduction in fecundity. Aphids feeding on transgenic plants expressing Mp-*AChE2* amiRs showed significantly more silencing of Mp-*AChE2* as compared to those feeding on hpRNA-expressing plant vectors for Mp-*AChE2*. Also the transgenics expressing Mp-*AChE2* amiRs showed improved insect resistance. The improved efficacy of Mp-*AChE2* amiRs over the hpRNA, could be due to the stability and the specificity of the amiRNAs compared to hpRNAs, which could be a better strategy for implementing RNAi *in planta*.

RNAi silencing of three gut-specific brown planthopper genes, hexose transporter gene *NlHT1*, carboxypeptidase gene *Nlcar*, and the trypsin-like serine protease gene *Nltry* in transgenic rice plants expressing dsRNA constructs failed to generate phenotypic changes in the insect (Zha et al., 2011). Third instar brown planthopper nymphs feeding on transgenic rice plants reduced the *NlHT1* and *Nlcar* transcript levels by about half in the midgut. However, such a significant reduction in the expression of target mRNA did not induce lethal phenotype, possibly due to either multiple copies of the target gene or limited changes at the protein level. In contrast, RNAi silencing of the abnormal wing disc (*Awd*) gene in Asian citrus psyllid had phenotypic effects (Hajeri et al., 2014). Pysillds feeding on citrus trees infected with recombinant Citrus tristeza virus (CTV) expressing *Awd*-silencing constructs had malformed wings and increased adult mortality. Gene expression analysis detected significant reduction in *Awd* transcripts in psyllids feeding on CTV-Awd infected citrus plants. The successful application of RNAi for Asian citrus psyllid control could significantly impact Huanglongbing (HLB) disease caused by the psyllid-vectored bacterial pathogen *Candidatus Liberibacter asiaticus* (CLas) (Hajeri et al., 2014).

Hemipteran insects readily develop resistance to pesticides, which could be overcome by targeting pesticide resistance genes using RNAi. The *carboxylesterase (CbE E4)* gene in grain aphids (*Sitobian avenae*) is responsible for developing resistance to a wide range of chemical pesticides that are routinely applied in agricultural fields (Xu et al., 2014). Grain aphids feeding on stable transgenic wheat plants expressing *CbE E4* dsRNA showed a 30–60% decrease in the *CbE E4* mRNA levels and reduced aphid numbers. Decreasing *CbE E4* gene expression could delay the development of resistance in this insect pest extending the utility of chemical management tools.

A novel method for *in planta* delivery of RNAi was tested for whiteflies by Luan et al. (2013). In separate experiments, uptake of dsRNA through the cut end of a tomato leaflet was accomplished by dipping petioles into solutions containing dsRNAs targeting whitefly genes *Cyp315a1* and *Cyp18a1*, involved in ecdysone 20E synthesis and degradation, respectively, or ecdysone response genes *EcR* and *E75*. In each of these treatments, silencing of these genes did not impact the survival and fecundity of the adult whiteflies. The exception was *EcR*-silenced adults, which laid fewer eggs. In all treatments, nymphs showed delayed development and poor survival rates (Luan et al., 2013).

Proof of principle for RNAi application in hemipteran insect control is demonstrated in these studies. However, successful deployment of RNAi technologies depends on the mode of delivery, effective dose, and target gene selection. *In planta* and spray delivery RNAi has potential for field applications, whereas microinjections and artificial diets are primarily limited to laboratory studies. As the cost of production of RNAi products become more economical, sprays, direct delivery of dsRNAs through plant cuttings or rooted seedlings, injecting trees and drip irrigation becomes more feasible (Hunter et al., 2012; Luan et al., 2013; Camargo et al., 2015).

## Cross-kingdom transfer of sRNAs

The ability to target insect genes by expressing dsRNAs in host plants provides compelling evidence for the cross-kingdom transfer of sRNAs; however, the role of endogenous plant-derived sRNAs directly impacting hemipteran insects has not been demonstrated. Phloem sap contains mobile sRNAs that are likely consumed by phloem-feeding hemipterans. Indeed, conserved plant miRNAs have been identified in phloem sap isolated by aphid stylectomy (Varkonyi-Gasic et al., 2010) and detected in aphid sRNA libraries (Sattar et al., 2012a). Direct evidence that sRNAs are readily consumed during normal feeding was demonstrated by aphids feeding on an artificial diet containing radio-labeled 24 nt dsRNA, which was detected in whole aphid tissues and in the honeydew excretia (Sattar et al., 2012a). However, the functional consequences for these dietary derived plant-sRNAs on the insect herbivore remains to be clarified (Cottrill and Chan, 2014; Witwer and Hirschi, 2014).

## Concluding remarks

Recent studies have recognized that sRNAs are important regulatory components of plant-hemipteran interactions. Within host plants, transcriptional changes in response to this unique form of insect herbivory are beginning to be correlated with concurrent changes in sRNA profiles. Co-expression networks and mRNA:sRNA interactomes are being assembled that are providing additional and sometimes unexpected information on the regulation of plant responses to insect herbivory. It is becoming increasingly clear that sRNAs are responsible for fine-tuning responses in a wide variety of plant-hemipteran interactions; however, unifying concepts for sRNA-mediated regulation across systems have yet to fully emerge. Understanding specific roles of sRNAs in host plant resistance along with advanced knowledge about the different components of the sRNA biosynthesis pathways can inform new pest control strategies for agricultural applications. Insects have co-evolved strategies to suppress plant immunity. Understanding these strategies, along with the contribution of insect sRNAs in regulating insect fitness and fecundity, provides additional insights that could allow sRNAs to be utilized in pest control. Insect anti-viral viRNAs that offer immunity against viral pathogens provide a new paradigm in understanding the complex plant-insect-virus interactions. The accumulation of viRNAs in response to virus acquisition leads to silencing of the viral genes, contributing to the vitality of the insect vector and its ability to infect new host plants. Emerging technologies based on our increasing knowledge of the role of sRNAs in regulating different aspect of plant-hemipteran interactions will greatly aid in developing next-generation alternatives to chemical pesticides. Ongoing work to identify and deliver effective RNAi approaches for hemipterans is paving the way for the rational design of target-specific pesticides that can complement current IPM techniques in the field.

## Author contributions

SS has done the literature search and SS and GT have written this manuscript.

### Conflict of interest statement

The authors declare that the research was conducted in the absence of any commercial or financial relationships that could be construed as a potential conflict of interest.
